# Melatonin Promotes Cheliped Regeneration, Digestive Enzyme Function, and Immunity Following Autotomy in the Chinese Mitten Crab, *Eriocheir sinensis*

**DOI:** 10.3389/fphys.2018.00269

**Published:** 2018-03-22

**Authors:** Cong Zhang, Xiao-zhen Yang, Min-jie Xu, Gen-yong Huang, Qian Zhang, Yong-xu Cheng, Long He, Hong-yu Ren

**Affiliations:** ^1^National Demonstration Center for Experimental Fisheries Science Education, Shanghai Ocean University, Shanghai, China; ^2^Key Laboratory of Freshwater Aquatic Genetic Resources, Ministry of Agriculture, Shanghai, China; ^3^Engineering Research Center of Aquaculture, Shanghai Ocean University, Shanghai, China

**Keywords:** *Eriocheir sinensis*, melatonin, eyestalk, regeneration, digestive enzyme, immunity

## Abstract

In the pond culture of juvenile *Eriocheir sinensis*, a high limb-impairment rate seriously affects the culture success. Therefore, it is particularly important to artificially promote limb regeneration. This study evaluated the effects of melatonin on cheliped regeneration, digestive ability, and immunity, as well as its relationship with the eyestalk. It was found that the injection of melatonin significantly increased the limb regeneration rate compared with the saline group (*P* < 0.05). The qRT-PCR results of growth-related genes showed that the level of *EcR-mRNA* (ecdysteroid receptor) and *Chi-mRNA* (chitinase) expression was significantly increased following the melatonin injection, while the expression of *MIH-mRNA* (molt-inhibiting hormone) was significantly decreased (*P* < 0.05). Melatonin significantly increased lipase activity (*P* < 0.05). We observed that the survival rates of limb-impaired and unilateral eyestalk-ablated crabs were substantially improved following melatonin treatment, whereas the survival of the unilateral eyestalk-ablated crabs was significantly decreased compared with the control group (*P* < 0.05). Furthermore, the results of serum immune and antioxidant capacity revealed that melatonin significantly increased the total hemocyte counts (THC), hemocyanin content, total antioxidant capacity (T-AOC), acid phosphatase (ACP), and glutathione peroxidase activity (GSH-Px), whereas the immune-related parameters were significantly decreased in eyestalk-ablated crabs (*P* < 0.05). Therefore, these findings indicate that melatonin exerts a protective effect on organism injury, which could promote limb regeneration by up-regulating the expression of growth-related genes, improve digestive enzyme activity, and strengthen the immune response, particularly antioxidant capacity.

## Introduction

Autotomy is an efficient reflexive response that is considered to be a useful adaptive mechanism for avoiding predators and limiting injuries, which commonly occur in both vertebrate and invertebrate populations (Wasson et al., 2002; Knope and Larson, 2014). Although autotomy can help animals temporarily escape from danger, the loss of one or more limbs can cause several adverse effects. For example, chelipeds play an important role in the defense, capture, manipulation, and subjugation of prey for crustaceans (Lawton, 1989). Moreover, cheliped autotomy may result in the loss of long-term energy and functionality (Fleming et al., 2007). Thus, the damage or loss of limbs and chelipeds can cause profound effects on feeding efficiency (Brock and Smith, 1998), immunity and antibacterial response (Yang et al., 2017), molting cycle (Quinitio and Estepa, 2011), survival rate (Simonson, 1985), and ecological community distribution (Oliveira et al., 2015). In addition, limb autotomy in the context of life- threatening situations typically serves as a temporary buffer. When an animal who has experienced limb-loss is attacked again, it is generally difficult for it to escape. Therefore, limb regeneration is the most effective means for crabs to compensate for the heavy cost of limb-loss.

The Chinese mitten crab, *Eriocheir sinensis*, is an important specialty aquaculture species, which occupies an important position in the Chinese aquaculture industry. In the pond culture of *E. sinensis*, various factors can cause a high rate of limb-loss (autotomy and impairment), including predation, fighting behavior, defense and foraging, lack of food, unsuccessful or unsynchronized molting, high-density farming, or artificial harvesting (Mcvean and Findlay, 1979; Lipcius and Herrnkind, 1982; Thomas et al., 2003; Riquelme-Bugueño, 2006; Rodriguez et al., 2007; Sui et al., 2011). Furthermore, a high rate of limb autotomy can seriously impact the culture benefit of *E. sinensis*. Therefore, it is of particular importance to artificially promote limb regeneration.

One study on the regulatory mechanisms of limb regeneration in *Uca pugilator* found that the process is primarily dependent on the co-regulation of ecdysteroid and molt-inhibiting hormone (MIH), in which ecdysteroid from the Y-organs and other tissues can promote regeneration. Moreover, since MIH produced in the eyestalk inhibits limb regeneration (Hopkins, 1992), in theory, eyestalk ablation can promote limb regeneration. However, there are no reports describing the effect of eyestalk on the limb regeneration of *E. sinensis*.

Melatonin is a well-known pineal hormone that participates in both circadian and seasonal rhythms and has been widely studied in vertebrates. Although melatonin has been found in almost all organisms, the role of melatonin in invertebrates is not fully understood. In crustaceans, melatonin is distributed throughout many tissues and organs, including the hemolymph, cranial ganglia, and eyestalk ganglia (Meyer-Rochow, 2001; Tilden et al., 2001; Pape et al., 2008). Moreover, some studies have demonstrated that melatonin has many significant physiological functions in crustaceans, such as regulating hemolymph glucose (Maciel et al., 2014), enhancing antioxidant capacity (Maciel et al., 2010), affecting reproductive function (Girish et al., 2015), promoting limb regeneration (Tilden et al., 1997), controlling the molting cycle, and mediating circadian rhythms (Sainath et al., 2013). However, the role of melatonin in the promotion of limb regeneration has not been studied in *E. sinensis*.

In crustaceans, ecdysteroid receptors (EcR) are activated by binding ecdysteroid (Hopkins et al., 2008). Moreover, chitinase (Chi) is crucial for crustacean molting and regeneration processes (Priya et al., 2009). Furthermore, digestive enzyme activity directly reflects the ability of an organism to digest and absorb nutrients, which determines the rate of growth and development (Lin et al., 2015). Although some studies have reported that eyestalk ablation or limb-impairment will affect crustacean immunity, the protective effect of melatonin on this type of injury has not been reported (Maggioni et al., 2004; Yang et al., 2017).

In the present study, we evaluated the cheliped regeneration rate (bud length R value), the expression of growth-related genes (*EcR, MIH*, and *Chi*), and the digestive enzyme activity of the hepatopancreas (activity of α-amylase, trypsin, and lipase) to verify the effect of melatonin on limb regeneration in *E. sinensis*. Moreover, we also evaluated the effects of melatonin on the survival rate of *E. sinensis* with cheliped autotomy and eyestalk-ablation, as well as the impact on immune function by measuring the changes in serum immune factors [total hemocyte counts (THC), hemocyanin content, and hydrolytic enzyme] and antioxidant capacity [total antioxidant capacity (T-AOC), superoxide dismutase (SOD), and glutathione peroxidase activity (GSH-Px)] during this process. Through the above experimental results, we initially explored the effects of melatonin on cheliped regeneration the mechanism of action, and potential relationship with the eyestalk.

## Materials and methods

### Experimental crabs

All experimental protocols were reviewed and approved by the Animal Bioethics Committee, Shanghai Ocean University, China. During early July 2017, 400 male hard-shelled crabs that had just completed molting and limb-intact *E. sinensis* (*Crustacea*; *Decapoda*; *Grapsidae*) juvenile crabs (4.98 g ± 0.90 g) were collected from a commercial farm in Jintan (Jiangsu Province, China). The juvenile crabs were acclimated for 1 week in monoculture systems that were supplied with continuous aerated freshwater [26–28°C; pH: 7.84 ± 0.08; dissolved oxygen (DO) concentration: 6.3 ± 0.4 mg/L; salinity: 0.3%, total ammonia: 0.36 ± 0.03 mg/L; chloride level: 136 ± 15 mg/L; and basal nitrite <0.05 mg/L^−1^] and natural photoperiod conditioning for 1 week. The crabs were fed once a day with a commercial crab diet.

### Experimental design

Healthy and limb-intact crabs were selected from the 400 crabs and subjected to autotomy of the left cheliped. Autonomy was achieved by gently grasping the limbs using the researcher's fingers, at which point, the crab would spontaneously autotomize the corresponding limb. The autotomized crabs were randomly divided into five groups (in triplicate): (1) Control group (C): autotomy of the left cheliped; (2) Saline group (S): a 20 μL injection of crustacean saline per crab (0.21 M NaCl, 13.6 mM KCl, 8.6 mM H_3_BO_3_, 4.75 mM NaOH, 20 mM MgSO_4_ · 7H_2_O; pH 7.2); (3) Unilateral eyestalk ablation group (UESA): unilateral eyestalk ablation was performed by clipping the left eyestalk using sterile scissors, and the wound was immediately cauterized to prevent the loss of hemolymph and avoid infection; (4) Melatonin injection group (MT): standard melatonin was purchased from Sigma-Aldrich Chemical company (USA) and dissolved in crustacean saline adjusted to a concentration of 10^−6^ mol/crab (absolute ethanol less than 1%), and 20 μL/crab was injected (Girish et al., 2015); (5) Unilateral eyestalk ablation + melatonin injection group (UESA+MT): following a unilateral eyestalk- ablation, the crabs were injected with 20 μL/crab standard melatonin solution. Prior to cheliped autotomy and eyestalk ablation, the crabs were anesthetized using ice, and both the crabs and experimental tools (i.e., scissors and tweezers) were disinfected with 75% alcohol cotton balls, respectively. The crabs were immediately returned to the aerated water in monoculture systems at the aquaculture environmental conditions described above.

### Sample collection

Crab cheliped buds were measured every 3 days for 12 days using a digimatic micrometer and dissecting microscope. The length of each cheliped bud was converted to a standardized *R*-value: *R* = (limb bud length/carapace width)^*^100 (Tilden et al., 1997). The experiment was completed after 2 weeks, and the survival rate of all groups was calculated at the end of the experiment. Ten individuals were randomly taken from each group for sample collection and were anesthetized on ice before each sampling. Hemolymph was drawn using a sterile 1-mL syringe from the unsclerotized membrane of the right third periopods and was immediately diluted 1:1 with sterile anticoagulant (30 mM trisodium citrate, 338 mM NaCl, 115 mM glucose, and 10 mM EDTA) for the total hemocyte counts (THC). The mixture was centrifuged at 42000 × g for 5 min, after which the serum was collected and stored at −20°C until the serum immune and antioxidant capacity could be evaluated. Hepatopancreas, epidermis, muscle, midgut, and hindgut samples were stored at −80°C for RNA isolation and further analysis using qRT-PCR to evaluate the level of *EcR, MIH*, and *Chi* gene expression. The remaining hepatopancreas samples were stored at −20°C for evaluation of digestive enzyme activity.

### Level of EcR, MIH, and chi gene expression using quantitative RT-PCR

Total RNA was extracted from the hepatopancreas, epidermis, muscle, midgut, and hindgut tissues using RNAiso™ plus reagent (RNA Extraction Kit, TaKaRa, Japan) according to the manufacturer's protocol. The concentration and quality of the total RNA were estimated using a micro-volume ultraviolet-visible spectrophotometer (Quawell Q5000; Thmorgan, China) and agarose-gel electrophoresis, respectively. The samples were reverse transcribed using the PrimeScript™ RT reagent Kit (Perfect Real Time, TaKaRa, Japan) according to the manufacturer's protocol. The obtained cDNA was diluted to 1:2 with double-distilled water and used as the qRT-PCR template. Relative quantification was performed using the ABI 7500 Real-Time PCR System (Life Technology, USA) with a SYBR® Premix Ex Taq™ (Tli RNaseH Plus, TaKaRa, Japan) kits using the following program: 95°C for 30 s; 40 cycles at 95°C for 5 s, 60°C for 34 s; followed by a melting curve at 95°C for 15 s, 60°C for 1 min, and 95°C for 15 s. The PCR primer sequences for *EcR, MIH*, and *Chi* are presented in Table 1 (Sangon Biotech Co. Ltd., Shanghai, China). β-actin was used as the internal control and performed in triplicate for each sample. Relative changes in the level of gene expression were determined by the 2^−ΔΔCt^ method. Data were analyzed and presented as the average values ± standard deviation (SD).

**Table 1:** Primer information for quantitative real-time polymerase chain reaction.

**Primers**	**Sequences (5′-3′)**
*EcR*-F	GGGCATCGGGCTACCACTACAAC
*EcR*-R	GGCACTGAGACTCGGGCACAACA
*MIH*-F	TGAAGACTGCGCCAACATCT
*MIH*-R	GCTCGTCAGGGTAGGTGGTG
*Chi*-F	GAGCCCTACGTCTACAGCATCAC
*Chi*-R	GGTCTCAACACTCCAAACCATCA
*β-actin*-F	TCATCACCATCGGCAATGA
*β-actin*-R	TTGTAAGTGGTCTCGTGGATG

### Evaluation of hepatopancreas digestive ability

The level of α-amylase, trypsin, and lipase activity was measured using a UV-spectrophotometer (Beijing Purkinje General Instrument Co., Ltd.) at 253, 660, and 420 nm with corresponding detection kits (Nanjing Jiancheng Bioengineering Institute, Nanjing, China) according to the manufacturer's protocol.

### Hemolymph sample analysis

#### Hemocyte THC levels

The levels of THC were obtained with a drop of the hemolymph anti-coagulant placed onto a hemocytometer using a Leica DMIL microscope (Leica Microsystems GmbH, Wetzlar, Germany). The count for each crab was repeated three times.

#### Immune-related parameters

Hemocyanin concentrations were determined using a UV-Spectrophotometric (Beijing Purkinje General Instrument Co., Ltd.) at 335 nm with 10 μL of serum diluted in 990 μL distilled water in a quartz cuvette, manually calibrated with distilled water. Hemocyanin concentrations (mmol/L) = 2.69 E (1%, 1 cm) mmol/L (Nickerson and Holde, 1971).

Acid phosphatase (ACP) and alkaline phosphatase (ALP) were measured using a UV-spectrophotometer (Beijing Purkinje General Instrument Co., Ltd.) at 520 nm with corresponding detection kits (Nanjing Jiancheng Bioengineering Institute, Nanjing, China) according to the manufacturer's protocols.

#### Anti-oxidant defense systems parameters

Commercial kits obtained for the total antioxidant capacity (T-AOC), superoxide dismutase (SOD), and glutathione peroxidase (GSH-Px) from Nanjing Jiancheng Bioengineering Institute (Nanjing, China) were used to measure their respective activity in the hemolymph supernatant. The activity of T-AOC (U/mL) (Fe^3+^ reduced to Fe^2+^ method), SOD (U/mL) (a method of superoxide anion radical (${\text{O}}_{2}^{-}$) oxidize hydroxylamine to form nitrite) and GSH-Px (μmol/L) (5, 5′-dithiobis-[2 -nitrobenzoic acid] method [DTNB]) were measured using a UV-spectrophotometer (Beijing Purkinje General Instrument Co., Ltd.) at 520, 550, 412 nm as described by the manufacturer's protocols, respectively.

### Statistical analyses

Data are presented as the average values of 10 replicates ± standard deviation (SD). In addition, the percentage values (dependent variable) were arcsine transformed before the analysis. A Tukey's Honestly Significant Difference Test (Tukey's HSD) and one-way ANOVA were used to analyze the statistical significance between the five groups, and a *P*-value < 0.05 was considered significant. All statistical analyses were performed using SPSS 20.0 software (Chicago, USA; Version 22.0).

## Results

### Cheliped regeneration

#### Cheliped bud length

**Figure 1:**
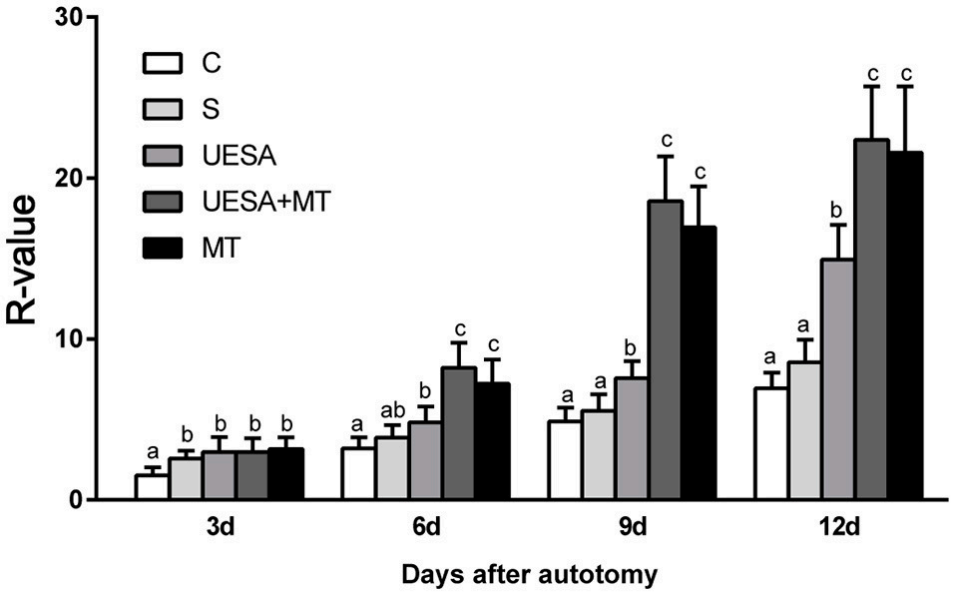
The cheliped bud length of *E. sinensis* over 12 days following the different treatments. The values are expressed as the means ± SD (*n* = 10). Different letters placed above column represent significant differences between treatments (*P* < 0.05).

Figure 1 shows the cheliped bud growth of the crabs over 12 days for the different treatment groups. The cheliped bud length was converted to a standardized *R*-value: *R* = (limb bud length /carapace width)^*^100. The R-value of the UESA group was significantly higher than that of the control group on Days 3, 6, 9, and 12. The cheliped bud length was significantly longer in MT group on Days 6, 9, and 12 compared to the saline and UESA groups on the same days. Moreover, the bud length of the UESA+MT group was significantly longer than that of other groups on Days 6, 9, and 12, with the exception that UESA+MT was not significantly from MT group.

#### The level of EcR, MIH, and Chi gene expression

**Figure 2:**
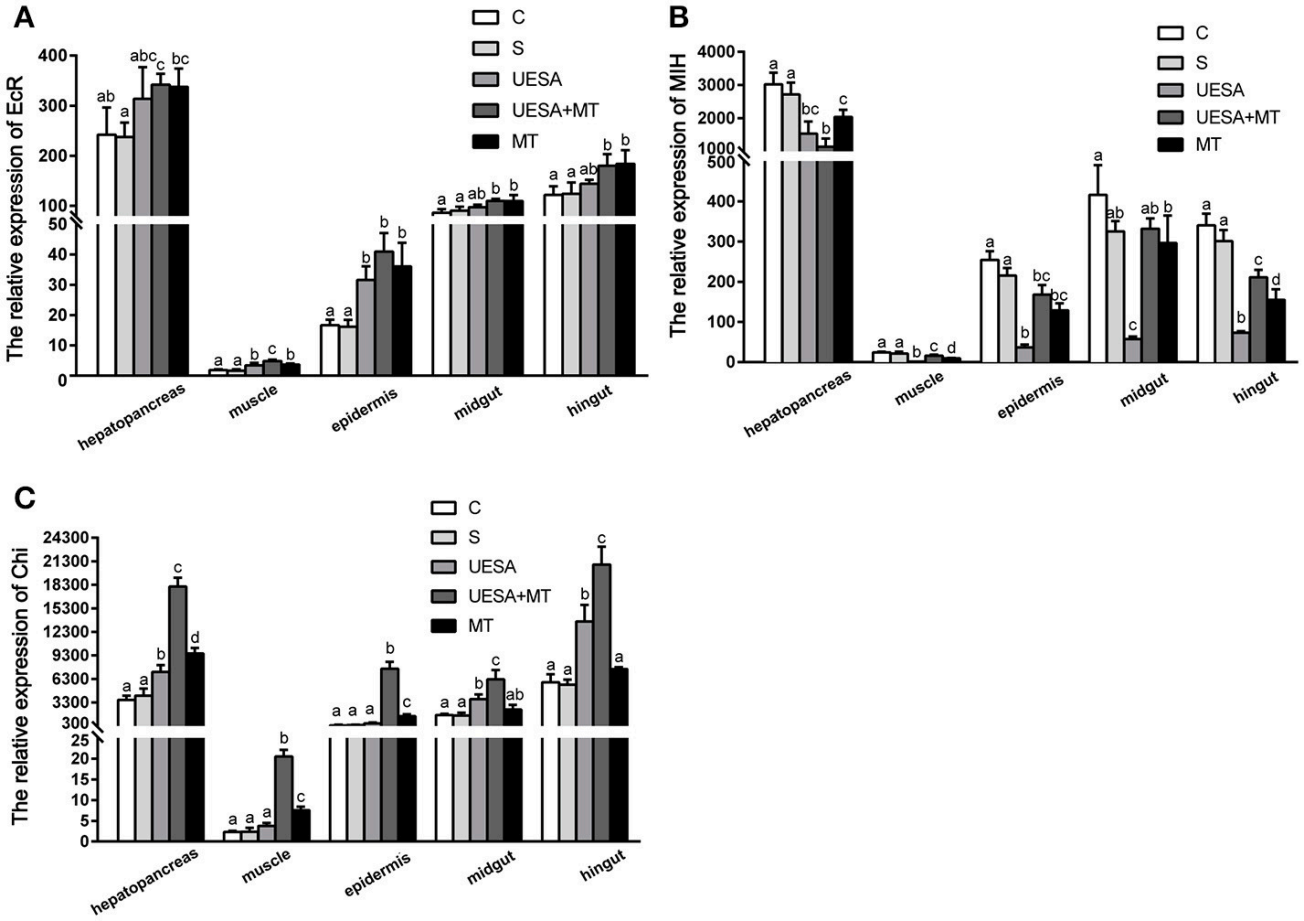
The level of *EcR, MIH*, and *Chi* gene expression normalized to β-actin in the hepatopancreas, muscle, epidermal, midgut, and hindgut of *E. sinensis* for each of the different treatments. **(A)** *EcR*, ecdysteroid receptor; **(B)** *MIH*, molt-inhibiting hormone; and **(C)** *Chi*, chitinase. The values are expressed as the means ± SD (*n* = 5). Different letters placed above column represent significant differences between treatments (*P* < 0.05).

To investigate the effect of the MT injection and eyestalk-ablation on regeneration-related gene expression during *E. sinensis* cheliped regeneration, the total RNA extracted from hepatopancreas, muscle, epidermis, midgut, and hindgut were subjected to quantitative real-time PCR using the primers pairs listed in Table 1. The results show a similar trend in all tissues (Figure 2). We observed that MT injection and/or unilateral eyestalk-ablation both caused a significant increase in *EcR-mRNA* expression in muscle and epidermis (*P* < 0.05); However, the expression of *EcR-mRNA* in the muscle was very low compared with the other tissues (Figure 2A).

The expression of *MIH-mRNA* was significantly decreased in the UESA and MT groups in the hepatopancreas, muscle, epidermal, and hindgut tissues compared with the crabs in the control and saline groups (*P* < 0.05) (Figure 2B).

The level of *Chi* gene expression exhibited a similar trend to that of *EcR* (Figures 2A,C). The level of Chi-mRNA expression was significantly increased in the MT group compared with the saline group in the hepatopancreas, muscle, and epidermal tissue (*P* < 0.05). The results also revealed that the level of *Chi-mRNA* expression in the UESA group was significantly increased compared with the control group in the hepatopancreas, midgut, and hindgut tissues (*P* < 0.05). Moreover, the level of *Chi-mRNA* expression of the crabs in the UESA+MT group was significantly higher than that in the tissues of all the other groups (*P* < 0.05) (Figure 2C).

### Digestive enzyme activity in the hepatopancreas

It was found that α-amylase activity was significantly improved by unilateral eyestalk-ablation (0.16 ± 0.01 U/mg prot) compared with the control group (0.12 ± 0.03 U/mg prot) (*P* < 0.05), whereas the highest level was observed in the UESA+MT group of trypsin activity (2.19 ± 0.07 U/mg prot), and it was significantly higher when compared to the other groups (*P* < 0.05). Moreover, lipase activity in the UESA group, UESA+MT group, and MT group (6.59 ± 0.74 U/mg prot, 6.18 ± 0.80 U/mg prot, 5.41 ± 0.64 U/mg prot, respectively) were significantly increased compared with that of the control group (4.07 ± 0.30 U/mg prot) and saline group (3.62 ± 0.54 U/mg prot) (*P* < 0.05) (Table 2).

**Table 2:** Effect of unilateral eyestalk ablation (UESA) and melatonin injection (MT) on the digestive enzymes activity in *E. sinensis*.

**Digestive enzymes (U/mgprot)**	**Groups**				
	**Control**	**Saline**	**UESA**	**UESA+MT**	**MT**
α-Amylase activity	0.12 ± 0.03^a^	0.14 ± 0.01^ab^	0.16 ± 0.01^b^	0.15 ± 0.01^ab^	0.13 ± 0.01^a^
Trypsin activity	1.85 ± 0.11^a^	1.81 ± 0.08^a^	1.79 ± 0.12^a^	2.19 ± 0.07^b^	1.91 ± 0.07^a^
Lipase activity	4.07 ± 0.30^a^	3.62 ± 0.54^a^	6.59 ± 0.74^b^	6.18 ± 0.80^b^	5.41 ± 0.64^b^

*The values are expressed as the means ± SD (n = 10). Different letters represent significant differences between treatments (P < 0.05)*.

### Survival rate and hemolymph analysis

#### Survival rate

**Figure 3:**
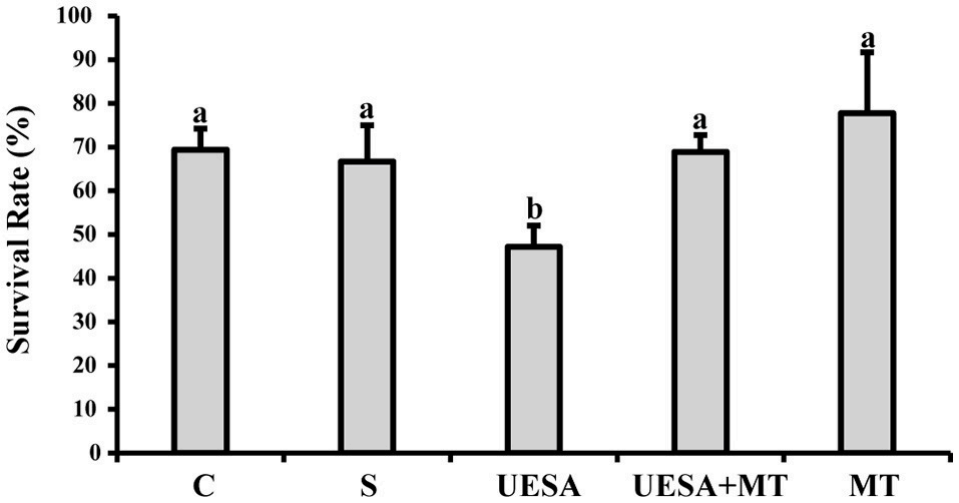
The survival rate of *E. sinensis* for all groups at the completion of the experiment. The values are expressed as the means ± SD (*n* = 3). Different letters placed above column represent significant differences between treatments (*P* < 0.05).

At the end of the experiment, we evaluated the survival rates of all groups as shown in Figure 3. The survival rate of the crabs in the UESA group (47.22 ± 4.81%) was significantly lower than that of the control group (69.44 ± 4.81%) (*P* < 0.05). However, the survival rate of the UESA+MT group (68.89 ± 3.85%) was significantly increased compared to that of the UESA group (*P* < 0.05). The UESA+MT survival in addition to being higher than the UESA group (*P* < 0.05) is also not significantly different from the control and saline groups. In addition, the survival rate of the MT group (77.78 ± 13.88%) was improved compared with that of the saline group (66.67 ± 8.33%), however, this improvement was not significant.

#### Total hemocyte counts (THC)

**Figure 4:**
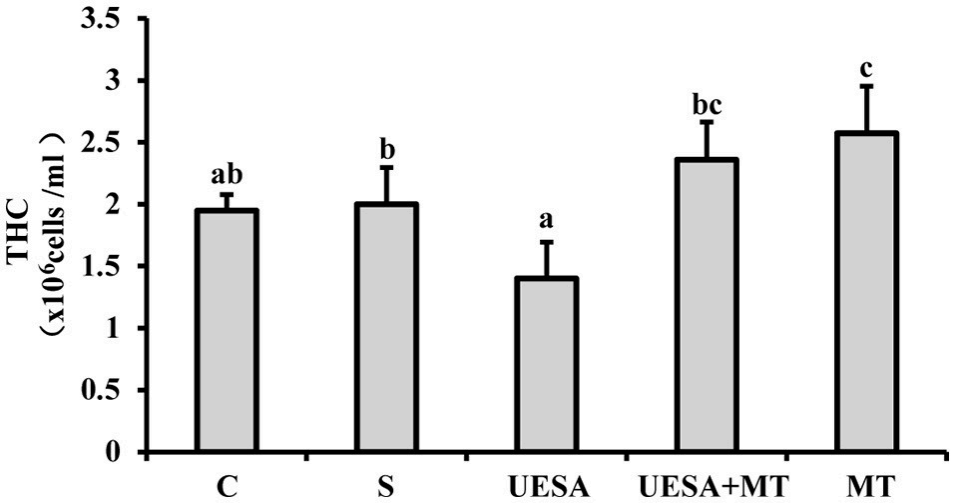
The level of THC in *E. sinensis* according to each treatment. The values are expressed as the means ± SD (*n* = 10). Different letters placed above column represent significant differences between treatments (*P* < 0.05).

Compared with the crabs in the control group (1.95 ± 0.13 × 10^6^ cells/mL), the level of THC exhibited a decreasing trend in the UESA group (1.40 ± 0.29 × 10^6^ cells/mL). However, the THC levels of the crabs in the UESA+MT group (2.36 ± 0.30 × 10^6^ cells/mL) were significantly higher compared with that of the UESA group (*P* < 0.05). These results also showed that the level of THC was significant higher in the MT group (2.58 ± 0.38 × 10^6^ cells/mL) compared to that of the saline (2.00 ± 0.30 × 10^6^ cells/mL; *P* < 0.05) and control groups *(P* < 0.05*)* (Figure 4).

#### Phosphatase activity and antioxidant capacity

**Figure 5:**
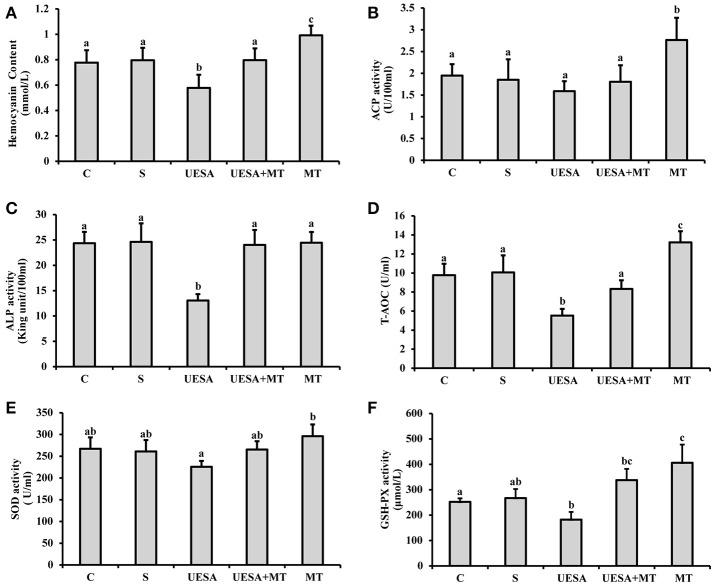
The effect of unilateral eyestalk ablation and melatonin injection on the immune and antioxidant capacity in *E. sinensis*. **(A)** Hemocyanin content; **(B)** acid phosphatase (ACP) activity; **(C)** alkaline phosphatase (ALP) activity; **(D)** total antioxidant capacity (T-AOC); **(E)** superoxide dismutase (SOD) activity; and **(F)** glutathione peroxidase (GSH-PX) activity. The values are expressed as the means ± SD (*n* = 10). Different letters placed above column represent significant differences between treatments (*P* < 0.05).

The hemocyanin content was significantly decreased in the UESA group (0.58 ± 0.10 mmol/mL), compared with that of the control group (0.78 ± 0.10 mmol/mL) (*P* < 0.05), whereas it was significantly increased in the UESA+MT group (0.80 ± 0.09 mmol/mL) compared with that of the UESA group *(P* < 0.05). Moreover, the results also showed that the hemocyanin content in the MT group (0.99 ± 0.08 mmol/mL) was significantly higher than that of the saline group (0.80 ± 0.10 mmol/mL) (*P* < 0.05) (Figure 5A).

ACP activity was significantly increased in the MT group (2.77 ± 0.51 U/100 mL), compared with that in the other groups (*P* < 0.05). In addition, unilateral eyestalk ablation (1.59 ± 0.23 U/100 mL) caused a decreasing trend in ACP activity compared to the control group (1.95 ± 0.26 U/100 mL). In contrast, after the crabs were unilaterally eyestalk-ablated, an injection with the standard melatonin solution (UESA+MT group: 1.80 ± 0.38 U/100 mL) increased ACP activity; however, the difference was not statistically significant (Figure 5B). Moreover, similar results were observed in that the ALP activity in the UESA group (13.06 ± 1.25 King unit/100 mL) was significantly lower than that of the other groups (*P* < 0.05). The ALP activity in the UESA+MT group (24.03 ± 2.94 King unit/100 mL) was significantly increased compared with that of the UESA group (*P* < 0.05) (Figure 5C).

T-AOC and GSH-PX activity in the UESA group (5.53 ± 0.71 U/mL and 182.4 ± 30.2 μmol/L, respectively) were significantly lower than that of the control group (9.77 ± 1.10 U/mL and 252.13 ±14.00 μmol/L, respectively) (*P* < 0.05) (Figures 5D,F). In contrast, T-AOC was significantly increased in the UESA+MT group (8.34 ± 0.90 U/mL) compared with that of the USEA group (*P* < 0.05). Moreover, T-AOC and GSH-PX activity in the MT group (13.22 ± 1.16 U/mL and 406.23 ± 71.42 μmol/L, respectively) were significantly higher than that of the saline group (10.06 ± 1.79 U/mL and 267.16 ± 35.57 μmol/L, respectively) (*P* < 0.05) (Figures 5D,F). Moreover, SOD activity in the MT group (296.08 ± 27.04 U/mL) was only significantly higher than that of the UESA group (225.80 ± 13.35 U/mL) (*P* < 0.05) (Figure 5E).

## Discussion

### Cheliped regeneration

#### Cheliped regeneration and growth-related gene expression

In nature, the limb autotomy phenomenon of crustaceans is extremely common, and is associated with several adverse effects, including the long-term loss of energy and functionality (Fleming et al., 2007), which affects feeding efficiency (Brock and Smith, 1998), and the distribution of ecological communities (Oliveira et al., 2015). Our previous study found that cheliped autotomy in *E. sinensis* caused a significant decrease in immunity and antibacterial ability (Yang et al., 2017). Moreover, in the pond culture of *E. sinensis*, limb autotomy seriously impacts quality and economic benefits. Therefore, it is of great significance to promote limb regeneration.

The limb regeneration process of crabs is performed under the joint regulation of ecdysteroid and molt-inhibiting hormone (MIH). Ecdysteroid is produced by Y-organs and other tissues and promotes limb regeneration, while MIH is produced from the eyestalk and can inhibit the release of ecdysteroid (Hopkins, 1992). Therefore, eyestalk ablation can reduce the release of molt-inhibiting hormone and promote limb regeneration. Tilden et al. (1997) reported that melatonin promotes the limb regeneration of *U. pugilator* and hypothesized that melatonin promoted the limb regeneration of crabs by inhibiting the release of MIH or directly stimulating the release of ecdysteroid (Tilden et al., 1997). In the present study, we found that consistent with the findings of Tilden et al. (1997), both unilateral eyestalk ablation and the injection of melatonin promoted limb regeneration. To investigate the mechanism by which melatonin promotes limb regeneration, we examined the expression of *EcR-mRNA* and *MIH-mRNA* in tissues of *E. sinensis* using qRT-PCR. The results revealed that unilateral eyestalk ablation significantly reduced the expression of *MIH-mRNA*, whereas *EcR-mRNA* expression was significantly increased compared to control and saline treatment groups. Similar results were observed in the MT injection group and UESA+MT group. However, the expression of *MIH-mRNA* in the MT injection group was higher than that of the unilateral eyestalk ablation group, and the expression of *EcR-mRNA* was significantly increased in the MT injection group. Girish et al. (2015) reported that an injection of melatonin caused a marked increase in the ecdysteroid levels of *Scylla serrata*, which was consistent with our results (Girish et al., 2015). Therefore, we speculate that melatonin inhibits the release of MIH and indirectly accelerates the expression of *EcR-mRNA*. Moreover, melatonin also directly stimulates the expression of *EcR-mRNA* to promote the limb regeneration of *E. sinensis*.

Limb regeneration of crabs is usually accompanied by a complete molting cycle. Since the limb regeneration process of crabs is relatively slow, the regeneration of new limbs is typically accompanied by the need to undergo a complete molting cycle (He et al., 2016). The molting activity of crustaceans is regulated by the promoting hormones (i.e., ecdysteroid and methyl farnesoate) and the inhibiting hormones (i.e., MIH and mandibular organ-inhibiting hormone [MOIH]). A scholar hypothesized that ecdysteroid and methyl farnesoate are secreted by the Y-organs and mandibular organ (MO), whereas MIH and MOIH are secreted by the X-organ-sinus gland (XO-SG) system in the eyestalk (Chang, 1993). Therefore, following eyestalk-ablation, the secretion of MIH decreased and the Y-organ was activated, compelling the Y-organs to secrete ecdysteroid, resulting in an increase of ecdysteroid in the hemolymph and accelerating the completion of molting cycle and limb regeneration. Similar studies have been reported in *Litopenaeus vannamei* (Sainz-Hernández et al., 2008), *Macrobrachium nipponense* (Sindhu Kumari, 1987), and *Penaeus monodon* (Nan et al., 1993).

Chitinase is crucial for both the crustacean molting and regeneration processes. Studies have shown that the binding of melatonin with the EcR and retinoid X receptor (RXR) positively regulate the expression of the chitinase gene in crustaceans (Priya et al., 2009). In the present study, we observed similar trends in *Chi-mRNA* and *EcR-mRNA* expression in the tissues of *E. sinensis*. Therefore, we concluded that melatonin and unilateral eyestalk-ablation could improve the expression of growth-promoting genes and facilitate the limb regeneration of *E. sinensis*.

#### Analysis of hepatopancreas digestive ability

Both unilateral eyestalk ablation and melatonin injection caused a significant increase in the expression of growth-related genes and accelerated the growth of *E. sinensis*. Therefore, we evaluated the effects of each of the different treatments on hepatopancreas digestive ability, and investigated the impact of eyestalk ablation and melatonin on digestibility. In crustaceans, the ability of the organism to digest and absorb nutrients can directly reflect the level of digestive enzyme activity, indicating an animal's status of growth and development (Girish et al., 2015; Lin et al., 2015). In the present study, unilateral eyestalk ablation significantly increased the level of α-amylase and lipase activity, whereas the trypsin activity did not change substantially. This is consistent with the findings of (Wu et al., 2013). In addition, the results of a study on *Palaemon serratus* showed that eyestalk ablation could significantly enhance digestive enzyme activity in the hepatopancreas (van Wormhoudt, 1974). In crustaceans, amylase and lipase are the main functional participants involved in the catabolism of carbohydrates and lipids. A previous study showed that unilateral eyestalk ablation caused carbohydrate and lipid content to significantly decrease in *Portunus sanguinolentus* (Sudhakar et al., 2009). Similarly, following eyestalk ablation in *Marsupenaeus japonicus*, the hepatopancreas index of parent shrimp decreased significantly, while glucose and triglyceride levels increased significantly in the hemolymph, indicating that eyestalk ablation accelerated the metabolism of carbohydrates and lipids in the body (Chao et al., 2009). Therefore, in this study, unilateral eyestalk ablation promoted the cheliped regeneration of *E. sinensis*, which consumed a large amount of energy. In addition, cheliped regeneration also requires a continued supply of energy; therefore, this process requires the hydrolysis and metabolism of additional carbohydrates and lipids, accelerating the metabolism, and recycling of materials, which is achieved by regulating the activity of amylase and lipase.

Moreover, similar results were also observed in the melatonin injection group. We found that a melatonin injection caused a significant increase in lipase activity in the hepatopancreas. Our observations in this study are consistent with the previous report that exogenous melatonin can prevent obesity in mice (Xu et al., 2017), reduces fat accumulation *in vivo*, and accelerates lipid metabolism. There was also a reported positive correlation between the level of melatonin and digestive enzyme activity, and digestive enzymes and gut melatonin exhibited an identical pattern of daily rhythms in *Catla catla* (Pal et al., 2016). Another study demonstrated that melatonin plays an important role in promoting intestinal digestion in *C. catla* (Maitra et al., 2015). Therefore, after the crabs were injected with melatonin, hepatopancreas lipase activity was increased, and the digestive and absorption ability of the crabs was enhanced, promoting growth, molting, and limb regeneration.

### Immunological analysis

It has been established that the survival rate and immune functionality of an organism are directly connected; when an organism's immune function is weakened, its survival rate is reduced. In the present study, we calculated the survival rate of each group throughout the experimental period, and found that unilateral eyestalk ablation significantly reduced the survival rate of *E. sinensis*. A similar result was also observed in *L. vannamei* with a unilateral or bilateral eyestalk ablation (Sainz-Hernández et al., 2008); however, interestingly, melatonin could significantly increase the survival rate of *E. sinensis* following a unilateral eyestalk ablation. Based on these results, we hypothesized that unilateral eyestalk ablation could weaken the immune system, whereas melatonin could enhance immunity and improve the anti-stress response by regulating the immune function of *E. sinensis*. Based on this point, the THC level, phosphatase activity, and antioxidant capacity were also investigated in the present study.

Several studies involving crustaceans have demonstrated that hematological parameters are important for assessing the immune response, including the level of THC, hydrolase activity, and antioxidant capacity (Cheng and Chen, 2000; Vogan and Rowley, 2002; Powell and Rowley, 2007). In the present study, we examined the effects of MT injection and unilateral eyestalk ablation on the hematological parameters of *E. sinensis* and subsequently assessed its effect on immune function. Studies have shown that THC is the most commonly used performance parameter for evaluating cellular immunity in crustaceans (van de Braak et al., 2002). In the current study, we observed that THC did not significantly decline following a unilateral eyestalk-ablation, whereas THC increased significantly following the melatonin injection. These results indicate that while the cellular immunity of the crab decreased following the unilateral eyestalk-ablation, a melatonin injection could improve this impairment. Similar results were also observed in female *L. vannamei* with unilateral eyestalk ablation (Maggioni et al., 2004).

Non-self-recognition protein, immunoglobulin, coagulation protein, antibacterial peptide, and other humoral factors play an important role in the immune defense of crustaceans (Lee and Söderhäll, 2002). In crustaceans, hemocyanin is an important multifunctional protein that primarily exists in the hemolymph, which accounts for more than 90% of the total protein in the serum (Lee et al., 2003; Zhang and Huang, 2004; Galindo et al., 2009). Hemocyanin plays several important roles, including carrying oxygen, transporting metal ions, storing proteins, osmoregulation, antibacterial activity, and the regulation of molting, which are all important functional participants in the immune system (Decker et al., 2007; Pan and Jin, 2008). In the present study, we observed that a unilateral eyestalk ablation significantly reduced hemocyanin levels in the serum, whereas the hemocyanin content returned to normal levels when melatonin was injected following a unilateral eyestalk-ablation. These results suggest that the unilateral eyestalk ablation reduced the immune function of *E. sinensis*, while the injection of melatonin enhanced the immunity of *E. sinensis*. In this study, a unilateral eyestalk ablation resulted in a significant decrease in the hemocyanin content, which is consistent with our previous studies on the effect of hemocyanin content following cheliped-ablation of *E. sinensis* (Yang et al., 2017). However, some studies have demonstrated that salinity (Wang et al., 2012) and hypoxia stress (Qin et al., 2016) can increase the hemocyanin content. These results indicate that the hemocyanin content was one of the evaluation indexes for the stress response of *E. sinensis*, and that different sources of stress have differential effects on the hemocyanin content.

ALP and ACP are two important non-specific phosphohydrolases that play an important role in the organism's self-defense system, and are considered to be important non-specific indicators of crustaceans, and reflect the overall health status of aquatic animals (Xue and Renault, 2000; Lin et al., 2011; Han et al., 2015). Thus, as the activities of ACP and ALP increase, cellular metabolism *in vivo*, non-specific immunity, and the rapid growth of the organism are improved. In the present study, unilateral eyestalk ablation resulted in a significant decrease in ALP activity, whereas the melatonin injection significantly increased ACP activity. These results imply that the unilateral eyestalk ablation of crabs reduced their non-specific immunity, and the injection of melatonin is able to enhance such immunity. Studies have shown that a melatonin injection in *Sparus aurata* L, increased immune-related gene expression (e.g., interleukin-1β) levels and non-specific immune immunoreactivity (i.e., phagocytosis), indicating that melatonin can enhance the immune response (Cuesta et al., 2008). Herrero et al. (2007) reported that increased plasma melatonin levels following dietary supplementation with melatonin reduced cortisol levels in *Dicentrarchus labrax*, thereby improving the anti-stress response (Herrero et al., 2007). In addition, Azpeleta et al. (2007) induced acute stress in goldfish by exposing them to air and found that this stress increased plasma cortisol levels, which could be reduced by melatonin injection group (Azpeleta et al., 2007). The results demonstrate that melatonin can improve the immune and anti-stress response by reducing cortisol levels.

The antioxidant defense system is also an important component of the crustacean immune defense system. T-AOC, superoxide dismutase (SOD), catalase (CAT), and glutathione peroxidase (GPH-Px) are important components of the crustacean antioxidant defense system (Zhao et al., 2013). When invertebrates are infected by pathogens, oxygen is released to enhance their resistance. An important indicator of crustacean cellular defense is the production of ROS, which is essential for normal cell function (e.g., redox signaling and anti-microbial responses); however, excessive ROS can induce oxidative damage, organ dysfunction, as well as DNA, protein, enzyme, and cell membrane destruction (Halliwell, 2006; Qin et al., 2016; Wu et al., 2017). SOD can convert harmful ${\text{O}}_{2}^{-}$ into hydrogen peroxide and then degrade the hydrogen peroxide product through GSH-Px to protect cells from oxidative damage (Holmblad and Söderhäll, 1999). In the present study, unilateral eyestalk ablation caused a significant decrease in GSH-Px and SOD activity in *E. sinensis*, while the injection of melatonin repaired the injury caused by unilateral eyestalk-ablation and the GSH-Px and SOD activity was significantly increased. These results indicate that melatonin can significantly improve the antioxidant capacity of *E. sinensis*. Moreover, T-AOC levels in the hemolymph of *E. sinensis* followed the same trend as GSH-Px and SOD. Several studies have emphasized that exogenous melatonin activity at pharmacological and physiological doses reduce oxidative stress by stimulating antioxidative enzymes (Barlow-Walden et al., 1995; Kotler et al., 1998; Trivedi and Jena, 2013). Baydas et al. (2002) also demonstrated that melatonin can directly or indirectly stimulate GSH-Px to reduce lipid peroxidation (Baydas et al., 2002). Furthermore, an injection of melatonin can significantly increase the immunity and antioxidant capacity of *Carassius auratus* (Jung et al., 2016), *Perdicula asiatica* (Kharwar and Haldar, 2012), and rats (Ozturk et al., 2000; Zhang et al., 2015). Therefore, our observations are consistent with previous reports that melatonin can enhance the immune defense of *E. sinensis*.

In conclusion, in the present study, we found that unilateral eyestalk ablation and a melatonin injection can promote limb regeneration by regulating the expression of growth-related genes and accelerate growth by enhancing hepatopancreas digestion. Moreover, unilateral eyestalk-ablation reduced the immune function of *E. sinensis*, whereas melatonin could enhance the body's antioxidant ability and immune defensive capacity. Our findings provide indications that the regulation the immune response in *E. sinensis* by melatonin may function at several levels of hematological immunity and antioxidant defense systems, rather than regulating the activity of a particular enzyme or immune-related protein. Moreover, unilateral eyestalk ablation did not inhibit the function of melatonin. Therefore, we speculate that melatonin can regulate immunity and limb regeneration through pathways other than the eyestalk in *E. sinensis*.

## Author contributions

X-zY designed the experiment and CZ wrote the article. M-jX, QZ, H-yR completed the determination of experimental indicators, G-yH and LH collected and analyzed data and Y-xC revised the article and made some positive suggestions.

### Conflict of interest statement

The authors declare that the research was conducted in the absence of any commercial or financial relationships that could be construed as a potential conflict of interest.

## References

[B1] Azpeleta C. De Pedro N. Velarde E. Delgado M. J. (2007). Melatonin attenuates the acute stress response in a teleost fish, *Carassius auratus*. Acta Physiol. 190, 118–122.

[B2] Barlow-Walden L. R. Reiter R. J. Abe M. Pablos M. Menendez-Pelaez A. Chen L. D.. (1995). Melatonin stimulates brain glutathione peroxidase activity. Neurochem. Int. 26, 497–502. 10.1016/0197-0186(94)00154-M 7492947

[B3] Baydas G. Gursu M. F. Yilmaz S. Canpolat S. Yasar A. Cikim G.. (2002). Daily rhythm of glutathione peroxidase activity, lipid peroxidation and glutathione levels in tissues of pinealectomized rats. Neurosci. Lett. 323, 195–198. 10.1016/S0304-3940(02)00144-1 11959418

[B4] Brock R. E. Smith L. D. (1998). Recovery of claw size and function following autotomy in *Cancer productus* (Decapoda: Brachyura). Biol. Bull. 194, 53–62. 10.2307/1542513 28574785

[B5] Chang E. S. (1993). Comparative endocrinology of molting and reproduction: insects and crustaceans. Annu. Rev. Entomol. 38, 161–180. 10.1146/annurev.en.38.010193.001113 8424625

[B6] Chao W. U. Lin Q. W. Zhang L. L. Shao-Jing L. I. Yuan G. S. Wen-Ping H. U. (2009). The influence of starvation and eyestalk ablation on the development of gonad and biochemical composition in hemolymph of *Marsupenaeus japonicus* Broodstock. J. Xiamen Univ. 2, 281–285. 10.3969/J.ISSN.2095-4972.2013.04.010

[B7] Cheng W. Chen J. C. (2000). Effects of pH, temperature and salinity on immune parameters of the freshwater prawn *Macrobrachium rosenbergii*. Fish Shellfish Immunol. 10, 387–391. 10.1006/fsim.2000.0264 10938749

[B8] Cuesta A. Cerezuela R. Esteban M. A. Meseguer J. (2008). *In vivo* actions of melatonin on the innate immune parameters in the teleost fish gilthead seabream. J. Pineal Res. 45, 70–78. 10.1111/j.1600-079X.2008.00557.x 18284550

[B9] Decker H. Hellmann N. Jaenicke E. Lieb B. Meissner U. Markl J. (2007). Minireview: recent progress in hemocyanin research. Integr. Comp. Biol. 47, 631–644. 10.1093/icb/icm063 21672868

[B10] Fleming P. A. Muller D. Bateman P. W. (2007). Leave it all behind: a taxonomic perspective of autotomy in invertebrates. Biol. Rev. Camb. Philos. Soc. 82, 481–510. 10.1111/j.1469-185X.2007.00020.x 17624964

[B11] Galindo C. Gaxiola G. Cuzon G. Chiappacarrara X. (2009). Physiological and biochemical variations during the molt cycle in Juvenile *Litopenaeus vannamei* under laboratory conditions. J. Crust. Biol. 29, 544–549. 10.1651/08-3094.1

[B12] Girish B. P. Ch S. Reddy P. S. (2015). Induction of ecdysteroidogenesis, methyl farnesoate synthesis and expression of ecdysteroid receptor and retinoid X receptor in the hepatopancreas and ovary of the giant mud crab, *Scylla serrata* by melatonin. Gen. Comp. Endocr. 217–218, 37–42. 10.1016/j.ygcen.2015.05.007 25989476

[B13] Halliwell B. (2006). Reactive species and antioxidants. Redox biology is a fundamental theme of aerobic life. Plant Physiol. 141, 312–322. 10.1104/pp.106.077073 16760481 PMC1475431

[B14] Han T. Wang J. Li X. Yang Y. Hu S. Jiang Y. (2015). Effects of dietary cholesterol levels on the growth, molt performance, and immunity of juvenile swimming crab, *Portunus trituberculatus*. Isr. J. Aquacult. Bamid 67, 1065–1069. Available online at: http://hdl.handle.net/10524/49203

[B15] He J. Wu X. Cheng Y. (2016). Effects of limb autotomy on growth, feeding and regeneration in the juvenile *Eriocheir sinensis*. Aquaculture 457, 79–84. 10.1016/j.aquaculture.2016.02.004

[B16] Herrero M. J. Martínez F. J. Míguez J. M. Madrid J. A. (2007). Response of plasma and gastrointestinal melatonin, plasma cortisol and activity rhythms of European sea bass (*Dicentrarchus labrax*) to dietary supplementation with tryptophan and melatonin. J. Comp. Physiol. B 177, 319–326. 10.1007/s00360-006-0131-6 17123089

[B17] Holmblad T. Söderhäll K. (1999). Cell adhesion molecules and an tioxidative enzymes in a crustacean, possible role in immunity. Aquaculture 172, 111–123. 10.1016/S0044-8486(98)00446-3

[B18] Hopkins P. Durica D. Washington T. (2008). RXR isoforms and endogenous retinoids in the fiddler crab, *Uca pugilator*. Comp. Biochem. Phys. A 151, 602–614. 10.1016/j.cbpa.2008.07.021 18692587

[B19] Hopkins P. M. (1992). Hormonal control of the molt cycle in the fiddler crab *Uca pugilator*. Am. Zool. 32, 450–458. 10.1093/icb/32.3.450

[B20] Jung S. J. Na N. K. Choi Y. J. Ji Y. C. Choi Y. U. Heo Y. S.. (2016). Effects of melatonin and green-wavelength LED light on the physiological stress and immunity of goldfish, *Carassius auratus*, exposed to high water temperature. Fish Physiol. Biochem. 42, 1335–1346. 10.1007/s10695-016-0221-7 27012684

[B21] Kharwar R. K. Haldar C. (2012). Daily variation in antioxidant enzymes and lipid peroxidation in lungs of a tropical bird *Perdicula asiatica* : role of melatonin and nuclear receptor RORα. Comp. Biochem. Phys. A 162, 296–302. 10.1016/j.cbpa.2012.01.021 22349119

[B22] Knope M. L. Larson R. J. (2014). Autotomy in porcelain crabs is an effective escape mechanism from rockfish predation. Mar. Ecol. 35, 471–477. 10.1111/maec.12103

[B23] Kotler M. Rodríguez C. Sáinz R. M. Antolín I. Menéndezpeláez A. (1998). Melatonin increases gene expression for antioxidant enzymes in rat brain cortex. J. Pineal Res. 24, 83–89. 10.1111/j.1600-079X.1998.tb00371.x 9510432

[B24] Lawton P. (1989). Predatory interaction between the brachyuran crab *Cancer pagurus* and decapod crustacean prey. Mar. Ecol. Prog. 52, 169–179. 10.3354/meps052169

[B25] Lee S. Y. Lee B. L. Söderhäll K. (2003). Processing of an antibacterial peptide from hemocyanin of the freshwater crayfish *Pacifastacus leniusculus*. J. Biol. Chem. 278, 7927–7933. 10.1074/jbc.M209239200 12493771

[B26] Lee S. Y. Söderhäll K. (2002). Early events in crustacean innate immunity. Fish Shellfish Immunol. 12, 421–437. 10.1006/fsim.2002.0420 12194453

[B27] Lin J. Zhang Q. Wensheng L. I. (2015). Comparison of the digestive enzyme activity of Nile tilapia (*Oreochromis niloticus*) in different farming modes. J. Fish. China 39, 65–74. 10.1016/j.anres.2017.04.005

[B28] Lin S. Pan Y. Luo L. Luo L. (2011). Effects of dietary β-1,3-glucan, chitosan or raffinose on the growth, innate immunity and resistance of koi (*Cyprinus carpio koi*). Fish Shellfish Immunol. 31, 788–794. 10.1016/j.fsi.2011.07.013 21784160

[B29] Lipcius R. N. Herrnkind W. F. (1982). Molt cycle alterations in behavior, feeding and diel rhythms of a decapod crustacean, the spiny lobster *Panulirus argus*. Mar. Biol. 68, 241–252. 10.1007/BF00409591

[B30] Maciel F. E. Geihs M. A. Cruz B. P. Vargas M. A. Allodi S. Marins L. F.. (2014). Melatonin as a signaling molecule for metabolism regulation in response to hypoxia in the crab *Neohelice granulata*. Int. J. Mol. Sci. 15, 22405–22420. 10.3390/ijms151222405 25486055 PMC4284716

[B31] Maciel F. E. Ramos B. P. Geihs M. A. Vargas M. A. Cruz B. P. Meyerrochow V. B.. (2010). Effects of melatonin in connection with the antioxidant defense system in the gills of the estuarine crab *Neohelice granulata*. Gen. Comp. Endocr. 165, 229–236. 10.1016/j.ygcen.2009.07.009 19607830

[B32] Maggioni D. S. Andreatta E. R. Hermes E. M. Barracco M. A. (2004). Evaluation of some hemato-immunological parameters in female shrimp *Litopenaeus vannamei* submitted to unilateral eyestalk ablation in association with a diet supplemented with superdoses of ascorbic acid as a form of immunostimulation. Aquaculture 241, 501–515. 10.1016/S0044-8486(03)00530-1

[B33] Maitra S. K. Mukherjee S. Hasan K. N. (2015). Melatonin: Endogenous Sources and Role in the Regulation of Fish Reproduction, Vol. 2. New York, NY: Nova Science Publishers Inc, 43–77.

[B34] Mcvean A. Findlay I. (1979). The incidence of autotomy in an estuarine population of the crab *Carcinus maenas*. J. Mar. Biol. Assoc. U.K. 59, 341–354. 10.1017/S0025315400042648

[B35] Meyer-Rochow V. B. (2001). The crustacean eye: dark/light adaptation, polarization sensitivity, flicker fusion frequency, and photoreceptor damage. Zool. Sci. 18, 1175–1197. 10.2108/zsj.18.1175 11911074

[B36] Nan F. H. Shyn-Shin S. Liu P. C. Chen S. N. (1993). The effect of eyestalk ablation on growth, haemolymph composition and gill Na^+^, K^+^ -ATPase activity of *Penaeus monodon* juveniles. Comp. Biochem. Phys. A 106, 621–626. 10.1016/0300-9629(93)90370-J

[B37] Nickerson K. W. Holde K. E. V. (1971). A comparison of molluscan and arthropod hemocyanin—I. Circular dichroism and absorption spectra. Comp. Biochem. Phys. B 39, 855–872. 10.1016/0305-0491(71)90109-X

[B38] Oliveira D. N. Christofoletti R. A. Barreto R. E. (2015). Feeding behavior of a crab according to cheliped number. PLoS ONE 10:e0145121. 10.1371/journal.pone.0145121 26682546 PMC4690604

[B39] Ozturk G. Coşkun S. Erbaş D. Hasanoglu E. (2000). The effect of melatonin on liver superoxide dismutase activity, serum nitrate and thyroid hormone levels. Jpn. J. Physiol. 50, 149–153. 10.2170/jjphysiol.50.149 10866707

[B40] Pal P. K. Hasan K. N. Maitra S. K. (2016). Temporal relationship between the daily profiles of gut melatonin, oxidative status and major digestive enzymes in carp *Catla catla*. Biol. Rhythm Res. 5, 755–771. 10.1080/09291016.2016.1191697

[B41] Pan L. Q. Jin C. X. (2008). A review on hemocyanins of crustacean. J. Fish. China 32, 484–491.

[B42] Pape C. Teschke M. Meyer B. (2008). Melatonin and its possible role in mediating seasonal metabolic changes of Antarctic krill, *Euphausia superba*. Comp. Biochem. Phys. A 149, 426–434. 10.1016/j.cbpa.2008.02.001 18328756

[B43] Powell A. Rowley A. F. (2007). The effect of dietary chitin supplementation on the survival and immune reactivity of the shore crab, Carcinus maenas. Comp. Biochem. Physiol. Part A Mol. Integr. Physiol. 147, 122–128. 10.1016/j.cbpa.2006.12.027 17289410

[B44] Priya T. A. Li F. Zhang J. Wang B. Zhao C. Xiang J. (2009). Molecular characterization and effect of RNA interference of retinoid X receptor (RXR) on E75 and chitinase gene expression in Chinese shrimp *Fenneropenaeus chinensis*. Comp. Biochem. Phys. B 153, 121–129. 10.1016/j.cbpb.2009.02.009 19250973

[B45] Qin F. Shi M. Yuan H. Yuan L. Lu W. Zhang J.. (2016). Dietary nano-selenium relieves hypoxia stress and, improves immunity and disease resistance in the Chinese mitten crab (*Eriocheir sinensis*). Fish Shellfish Immunol. 54, 481–488. 10.1016/j.fsi.2016.04.131 27153751

[B46] Quinitio E. T. Estepa F. D. P. (2011). Survival and growth of Mud crab, *Scylla serrata*, juveniles subjected to removal or trimming of chelipeds. Aquaculture 318, 229–234. 10.1016/j.aquaculture.2011.05.034

[B47] Riquelme-Bugueño R. (2006). Incidence patterns of limb autotomy in the estuarine crab, *Hemigrapsus crenulatus* (H. Milne Edwards, 1837) (Brachyura, Grapsoidea) from a temperate estuary in the Eastern South Pacific. Crustaceana 79, 925–932. 10.1163/156854006778815973

[B48] Rodriguez E. M. Parado-Estepa F. D. Quinitio E. T. (2007). Extension of nursery culture of *Scylla serrata* (Forsskål) juveniles in net cages and ponds. Aquac. Res. 38, 1588–1592. 10.1111/j.1365-2109.2007.01725.x

[B49] Sainath S. B. Ch S. Reddy P. S. (2013). What do we (need to) know about the melatonin in crustaceans? J. Exp. Zool. A 319, 365–377. 10.1002/jez.1800 23650247

[B50] Sainz-Hernández J. C. Racotta I. S. Dumas S. Hernández-López J. (2008). Effect of unilateral and bilateral eyestalk ablation in *Litopenaeus vannamei* male and female on several metabolic and immunologic variables. Aquaculture 283, 188–193. 10.1016/j.aquaculture.2008.07.002

[B51] Simonson J. L. (1985). Reversal of handedness, growth, and claw stridulatory patterns in the stone crab *Menippe mercenaria* (Say) (Crustacea: Xanthidae). J. Crust. Biol. 5, 281–293. 10.2307/1547875

[B52] Sindhu Kumari S. (1987). Effects of unilateral eyestalk ablation on moulting, growth, reproduction and energy budget of *Macrobrachium nobili*. Asian Fish. Sci. 1, 1–17.

[B53] Sudhakar M. Manivannan K. Soundarapandian P. Ananthan G. (2009). Effect of unilateral eyestalk ablation on the biochemical changes of edible portunidae crab *Portunus sanguinolentus* (HERBST). Middle East J. Sci. Res. 4, 153–157. 10.1099/0022-1317-11-1-35

[B54] Sui L. Wille M. Cheng Y. Wu X. Sorgeloos P. (2011). Larviculture techniques of Chinese mitten crab *Eriocheir sinensis*. Aquaculture 315, 16–19. 10.1016/j.aquaculture.2010.06.021

[B55] Thomas C. W. Carter C. G. Crear B. J. (2003). Feed availability and its relationship to survival, growth, dominance and the agonistic behaviour of the southern rock lobster, *Jasus edwardsii* in captivity. Aquaculture 215, 45–65. 10.1016/S0044-8486(01)00899-7

[B56] Tilden A. R. Alt J. Brummer K. Groth R. Herwig K. Wilson A.. (2001). Influence of photoperiod on N-acetyltransferase activity and melatonin in the fiddler crab *Uca pugilator*. Gen. Comp. Endocr. 122, 233–237. 10.1006/gcen.2001.7641 11356035

[B57] Tilden A. R. Rasmussen P. Awantang R. M. Furlan S. Goldstein J. Palsgrove M.. (1997). Melatonin cycle in the fiddler crab *Uca pugilator* and influence of melatonin on limb regeneration. J. Pineal Res. 23, 142–147. 10.1111/j.1600-079X.1997.tb00347.x 9406985

[B58] Trivedi P. P. Jena G. B. (2013). Melatonin reduces ulcerative colitis-associated local and systemic damage in mice: investigation on possible mechanisms. Dig. Dis. Sci. 58, 3460–3474. 10.1007/s10620-013-2831-6 23975342

[B59] van de Braak C. B. Botterblom M. H. Liu W. Taverne N. van der Knaap W. P. Rombout J. H. (2002). The role of the haematopoietic tissue in haemocyte production and maturation in the black tiger shrimp (*Penaeus monodon*). Fish Shellfish Immunol. 12, 253–272. 10.1006/fsim.2001.0369 11931020

[B60] van Wormhoudt A. (1974). Variations of the level of the digestive enzymes during the intermolt cycle of *Palaemon serratus*: influence of the season and effect of the eyestalk ablation. Comp. Biochem. Phys. A 49, 707–715. 10.1016/0300-9629(74)90899-8 4154172

[B61] Vogan C. L. Rowley A. F. (2002). Effects of shell disease syndrome on the haemocytes and humoral defences of the edible crab, *Cancer pagurus*. Aquaculture 205, 237–252. 10.1016/S0044-8486(01)00703-7

[B62] Wang Y. R. Er-Chao L. I. Chen L. Q. Wang X. D. Zhang F. Y. Gao L. J. (2012). Effect of actute salinity stress on soluble protein, hemocyanin, haemolymph glucose and hepatopancreas glycogen of *Eriocheir sinensis*. Acta Hydrobiol. Sin. 36, 1056–1062. 10.3724/SP.J.1035.2012.01056

[B63] Wasson K. Lyon B. E. Knope M. (2002). Hair-trigger autotomy in porcelain crabs is a highly effective escape strategy. Behav. Ecol. 13, 481–486. 10.1093/beheco/13.4.481

[B64] Wu J. L. Kang X. J. Mu S. M. Tian Z. H. (2013). Effect of eyestalk ablation in *Eriocheir sinensis* on physiological and biochemical metabolism. Agric. Sci. 4, 25–29. 10.4236/as.2013.46A004

[B65] Wu Y. S. Lee M. C. Huang C. T. Kung T. C. Huang C. Y. Nan F. H. (2017). Effects of traditional medical herbs “minor bupleurum decoction” on the non-specific immune responses of white shrimp (*Litopenaeus vannamei*). Fish Shellfish Immunol. 64, 218–225. 10.1016/j.fsi.2017.03.018 28288911

[B66] Xu P. Wang J. Hong F. Wang S. Jin X. Xue T.. (2017). Melatonin prevents obesity through modulation of gut microbiota in mice. J. Pineal Res. 62:e12399. 10.1111/jpi.12399 28199741

[B67] Xue Q. G. Renault T. (2000). Enzymatic activities in European flat oyster, *Ostrea edulis*, and Pacific oyster, *Crassostrea gigas*, hemolymph. J. Invertebr. Pathol. 76, 155–163. 10.1006/jipa.2000.4965 11023742

[B68] Yang X. Z. Zhang C. Huang G. Y. Xu M. J. Cheng Y. X. Yang Z. G.. (2017). Cellular and biochemical parameters following autotomy and ablation-mediated cheliped loss in the Chinese mitten crab, *Eriocheir sinensis*. Dev. Comp. Immunol. 81, 33–43. 10.1016/j.dci.2017.11.003 29146453

[B69] Zhang L. Gong J. T. Zhang H. Q. Song Q. H. Xu G. H. Cai L.. (2015). Melatonin attenuates noise stress-induced gastrointestinal motility disorder and gastric stress ulcer: role of gastrointestinal hormones and oxidative stress in rats. J. Neurogastroenterol. 21, 189–199. 10.5056/jnm14119 25537679 PMC4398253

[B70] Zhang X. Huang C. Q. (2004). Antiviral properties of hemocyanin isolated from shrimp *Penaeus monodon*. Antivir. Res. 61, 93–99. 10.1016/j.antiviral.2003.08.019 14670582

[B71] Zhao Y. T. Xu-Gan W. U. Chang G. L. Qiu R. J. Cheng Y. X. (2013). Effects of dietary DHA Levels on growth, lipid composition and hypoxia stress of juvenile Chinese mitten crab *Eriocheir sinensis*. Acta Hydrobiol. Sin. 37, 1133–1144. 10.7541/2013.154

